# Measurement Biases Distort Cell-Free DNA Fragmentation Profiles and Define the Sensitivity of Metagenomic Cell-Free DNA Sequencing Assays

**DOI:** 10.1093/clinchem/hvab142

**Published:** 2021-10-26

**Authors:** Adrienne Chang, Omary Mzava, Joan S Lenz, Alexandre P Cheng, Philip Burnham, S Timothy Motley, Crissa Bennett, John T Connelly, Darshana M Dadhania, Manikkam Suthanthiran, John R Lee, Amy Steadman, Iwijn De Vlaminck

**Affiliations:** 1 Nancy E. and Peter C. Meinig School of Biomedical Engineering, Cornell University, Ithaca, NY, USA; 2 Global Good Fund, Intellectual Ventures Lab, Bellevue, WA, USA; 3 Global Health Labs, Bellevue, WA, USA; 4 Division of Nephrology and Hypertension, Department of Medicine, Weill Cornell Medicine, New York, NY, USA; 5 Department of Transplantation Medicine, New York Presbyterian Hospital, Weill Cornell Medical Center, New York, NY, USA

**Keywords:** cell-free DNA, pre-analytical, liquid biopsy

## Abstract

**Background:**

Metagenomic sequencing of microbial cell-free DNA (cfDNA) in blood and urine is increasingly used as a tool for unbiased infection screening. The sensitivity of metagenomic cfDNA sequencing assays is determined by the efficiency by which the assay recovers microbial cfDNA vs host-specific cfDNA. We hypothesized that the choice of methods used for DNA isolation, DNA sequencing library preparation, and sequencing would affect the sensitivity of metagenomic cfDNA sequencing.

**Methods:**

We characterized the fragment length biases inherent to select DNA isolation and library preparation procedures and developed a model to correct for these biases. We analyzed 305 cfDNA sequencing data sets, including publicly available data sets and 124 newly generated data sets, to evaluate the dependence of the sensitivity of metagenomic cfDNA sequencing on pre-analytical variables.

**Results:**

Length bias correction of fragment length distributions measured from different experimental procedures revealed the ultrashort (<100 bp) nature of microbial-, mitochondrial-, and host-specific urinary cfDNA. The sensitivity of metagenomic sequencing assays to detect the clinically reported microorganism differed by more than 5-fold depending on the combination of DNA isolation and library preparation used.

**Conclusions:**

Substantial gains in the sensitivity of microbial and other short fragment recovery can be achieved by easy-to-implement changes in the sample preparation protocol, which highlights the need for standardization in the liquid biopsy field.

The observation that microbial cell-free DNA (cfDNA) is present in biofluids has inspired new avenues for infectious disease testing (1). Recent studies have demonstrated the utility of cfDNA metagenomic sequencing of blood and urine to detect a wide range of pathogens that cause a variety of complications, including urinary tract infection, blood-borne infection, and deep-seated infection of tissues that would otherwise require invasive biopsies for diagnosis (2–13). The sensitivity of metagenomic cfDNA sequencing is partly determined by the efficiency of recovery of microbial vs host cfDNA. We reasoned that the choice of DNA isolation and library preparation methods would strongly affect the sensitivity of metagenomic cfDNA sequencing because the yield of DNA isolation and library preparation protocols depends on the physical length of the assayed DNA, and because microbial cfDNA is more fragmented than host-specific cfDNA (14). In this study, we characterized the fragment length biases inherent to select DNA isolation and library preparation procedures and developed a model to correct for these biases. Our study demonstrates that substantial gains in microbial and other short fragment recovery can be obtained by easy-to-implement changes in the sample preparation protocol and highlights the need for standardization in the liquid biopsy field.

## Materials and Methods

### Synthetic cfDNA Preparation and Transfer Function Calculations

**Fig. 1.**
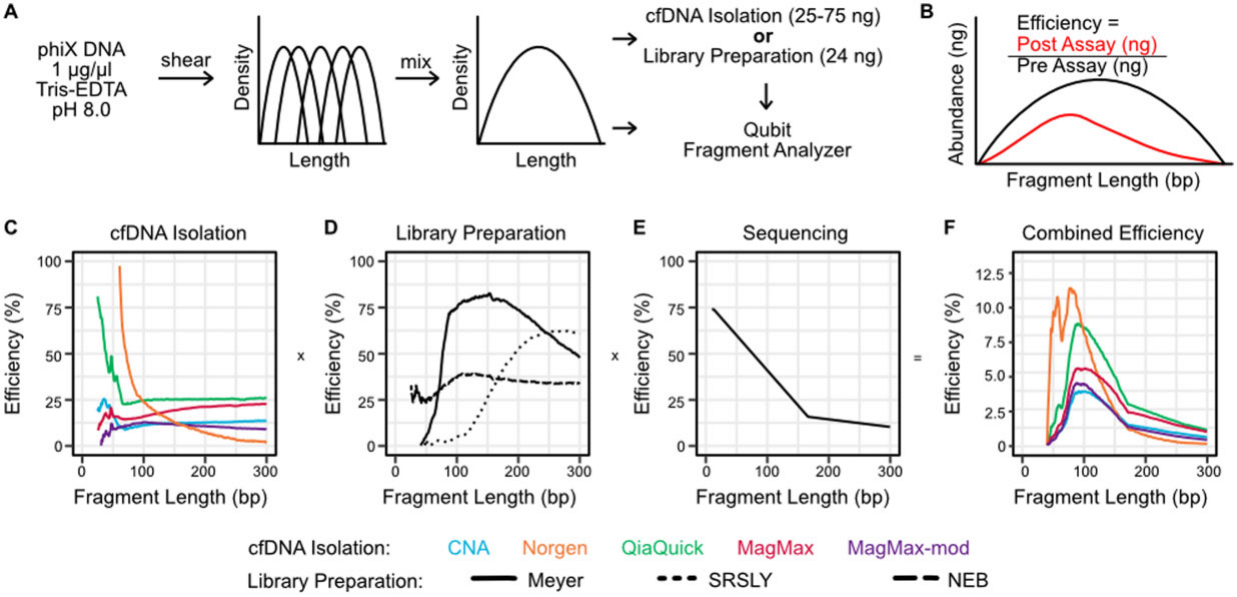
Characterization of fragment length bias introduced by cfDNA sequencing assays. **(**A) Synthetic cfDNA was prepared by mixing sheared ΦX174 DNA. (B) The fragment length distributions were measured before and after each assay step to obtain the efficiency of fragment recovery as a function of fragment length. The product of (C) cfDNA isolation, (D) library preparation, and (E) sequencing efficiencies was taken to be (F) the overall transfer function (shown for various isolation kits after library preparation with the Meyer protocol).

Synthetic cfDNA was prepared by shearing (E2220evolution, Covaris) ΦX174 RF I DNA (NEB) at a concentration of 1 μg/μL in Tris-EDTA, pH 8.0, to 5 target fragment lengths (150, 200, 300, 400, and 500 bp) and mixed such that the resulting fragment length profile exhibited a smooth, broad distribution (Fig. 1, A ). Samples for cfDNA isolation assays were prepared by diluting 25, 50, or 75 ng of synthetic cfDNA into 1 mL of artificial urine pH 6.6 (Pickering Laboratories) such that the resulting solution reflected the concentration and chemical composition of urinary cfDNA (15). For library preparation assays, 24 ng of synthetic cfDNA in 10 mM Tris-HCl, pH 8.0, was added per sample. All assays were immediately conducted following sample preparation to ensure DNA fidelity (16,17).

Samples were quantified using the Qubit dsDNA HS Assay (Qubit Fluorometer 3.0, Invitrogen) and characterized using the AATI Fragment Analyzer HS NGS Fragment Kit (Agilent) before and after each assay. Length traces were adjusted to account for library preparation adapter sequences and normalized using the bayestestR package (method = “trapezoid”) (16,17). The normalized trace was scaled by the input biomass to obtain the abundance profile. The experimental efficiency for each assay (n = 15 replicates per assay) was calculated as a function of the fragment length as:

The transfer function for each unique workflow combination of cfDNA isolation, library preparation, and sequencing [obtained from (18)] was produced by multiplying the experimental efficiency curve for each step.

### Study Cohort and Sample Collection

One hundred and forty-two urine samples were collected from 83 kidney transplant recipients who received their transplant at New York Presbyterian Hospital-Weill Cornell Medical Center (kidney transplant patients), with 30 of these samples evaluated using different cfDNA isolation or library preparation methods (11). This study was approved by the Weill Cornell Medicine Institutional Review Board (protocols 9402002786, 1207012730, 0710009490). Additionally, 66 samples were collected from individuals seeking tuberculosis treatment through a study partly funded by the Department of Science and Technology—Philippine Council for Health Research and Development (DOST-PCHRD, tuberculosis patients). This study was approved by the University of the Philippines Manila Research Ethics Board (protocol UPMREB 2018-252-01).

Samples were collected via the conventional method for a clean-catch midstream specimen used for standard urine culture. For the kidney transplant patient samples, approximately 50 mL of urine was centrifuged at 3000*g* on the same day for 30 min and the supernatant was stored in 1 mL aliquots at −80°C. For the tuberculosis patient samples, 10 mL of urine was mixed with 2 mL Streck cell-free DNA urine preserve (Streck, USA) and centrifuged at 3000*g* for 30 min at ambient temperature. The supernatant was similarly stored in 1 mL aliquots at −80°C.

Additionally, 40 plasma samples from 6 individuals receiving double-lung transplants at Stanford University Hospital collected in the scope of a previous study were included (lung transplant recipients) (1, 12). Briefly, peripheral blood was collected in EDTA tubes and centrifuged at 16 000*g* for 10 min within 24 h after blood collection. Plasma was stored in 1 mL aliquots at −80°C. All individuals provided written informed consent.

### cfDNA Isolation and Library Preparation Assays

To evaluate the performance of different kits for various downstream applications, we analyzed the performance of spin column- and magnetic bead-based commercial kits targeted for cfDNA or short fragment isolation. Urinary cfDNA and synthetic cfDNA samples were isolated and libraries were prepared using the kits detailed in Table 1. All kits were used according to the manufacturer’s instructions, except for a modified MagMax protocol described below.

**Table 1:** cfDNA isolation and library preparation kits compared in this study.

Kit type	Name	Code	Principle
cfDNA isolation	QIAamp Circulating Nucleic Acid Kit (Qiagen)	CNA	Spin-column
cfDNA isolation	Urine Cell-Free Circulating DNA Purification Kit (Norgen)	Norgen	Spin-column
cfDNA isolation	QIAquick Nucleotide Removal Kit (Qiagen)	QiaQuick	Spin-column
cfDNA isolation	MagMAX Cell-Free DNA Isolation Kit (Thermo Fisher Scientific), per manufacturer’s guidelines	MagMax	Magnetic bead
cfDNA isolation	MagMAX Cell-Free DNA Isolation Kit (Thermo Fisher Scientific), modified protocol	MagMax-mod	Magnetic bead
Library preparation	Meyer [Burnham et al. (14), Gansauge et al. (19)]	Meyer	Single-stranded
Library preparation	SRSLY NGS Library Prep Kit (Claret Bioscience)	SRSLY	Single-stranded
Library Preparation	NEBNext Ultra II DNA Library Prep Kit for Illumina (New England Biolabs)	NEB	Double-stranded

The Qiagen CNA kit was chosen as the gold standard reference for the cfDNA isolation assays because it was previously shown to be the most consistent and efficient cfDNA isolation kit (20–24). The Norgen kit was chosen because it was recently shown to outperform the CNA kit in DNA recovery (25). While the QiaQuick kit is not advertised for cfDNA isolation, it was included in this study because it was designed to retain small oligonucleotides of lengths 17 to 40 bp. Finally, the MagMax kit was included as it is the most common magnetic bead-based isolation kit employed in cfDNA studies. Additionally, a modified version of the MagMax protocol was included because it was shown to improve the isolation of short fragments (data not shown). The following changes to the MagMax protocol were made:

1. The extraction master mix was made with 1.25 mL MagMax lysis/binding solution, 0.5 mL isopropanol, and 7.5 μL MagMax beads per 1 mL sample.
2. After combining the appropriate volumes of extraction mix and urine, samples were rotated on a rotisserie inverter for 30 min at room temperature.
3. The cfDNA was eluted from beads in 50 μL MagMax DNA elution solution prewarmed to 55°C by vortexing in a heated shaker at 1400 rpm, 55°C for 20 min.
4. The second bind–wash–elute steps were removed from the workflow.

Single -stranded sequencing libraries were prepared either as previously described (14) or using the SRSLY kit. Double-stranded sequencing libraries were prepared using the NEB kit. All library preparations followed the manufacturer’s protocol and were pooled and sequenced (paired-end, 2 x 75 bp) on the NextSeq 500 platform (Illumina).

### Measuring the Fragment Length Distributions, Intrinsic Densities, and Microbial Enrichment

Low-quality bases and Illumina-specific sequences were trimmed (Trimmomatic-0.32, LEADING : 25 TRAILING : 25 SLIDINGWINDOW : 4:30 MINLEN : 15) (26). Reads were aligned (BWA-mem) (27) to the human reference (UCSC hg19). Reads that did not align to the human reference were extracted and aligned to the circularized *Mycobacterium tuberculosis H37Rv* (edited from NC_000962.3) and the circularized *Escherichia coli* (edited from NZ_CP027599.1) genomes. The lengths for reads aligning to the chromosomal, mitochondrial, and microbial genomes with a minimum mapping quality of 30 and minimum insert size of 45 bp were calculated as the absolute difference between the start and end coordinates of the mapped read. The measured fragment length density profile was computed using the *hist* function from the R graphics package for lengths 25 to 500 bp.

To compute the intrinsic fragment length profile, the measured distributions were first scaled to the input biomass and then multiplied with the relevant transfer function across all fragment lengths. The convergence of the cumulative distributions was evaluated using the *ks.test* function in the standard stats package in R.

Samples with culture-confirmed urinary tract infection were chosen to evaluate the effects of cfDNA isolation and library preparation on microbial enrichment. Two aliquots from each of 10 samples were subject to isolation using the CNA kit in combination with either the NEB or SRSLY library preparation kit. An additional 2 aliquots from another set of 20 samples were each subject to a unique cfDNA isolation kit followed by library preparation using the Meyer protocol (14). All the samples were previously prepared by isolation with the CNA kit followed by the Meyer protocol (Table 2), which enabled us to utilize the CNA and Meyer protocols as a gold standard reference. Non -human reads were aligned to an annotated National Center for Biotechnology Information (NCBI) microbial database using BLAST (28) and the relative genome abundance was calculated using GRAMMy (29). The molecules of microbial cfDNA per million reads (MPM) was computed as the ratio of culture-identified microbial vs non-duplicate host reads in the sample. The non-duplicate host reads were used to account for operator-to-operator variability in library preparation and differences in sequencing depth.

**Table 2:** Overview of included data sets. All data were generated for this study, unless otherwise indicated. Figures that include the specified samples are indicated.

Cohort	Biofluid	Combination of methods used^*^	Figures
Kidney transplant recipients	Urine	CNA + Meyer = 142 [Burnham et al. (11)]	Figs. 2 and 3
		CNA + SRSLY = 10	Fig. 3, B
		CNA + NEB = 10	Fig. 3, B
		Norgen + Meyer = 10	Figs. 2, A and 3, A
		QiaQuick + Meyer = 9	Figs. 2, A and 3, A
		MagMax + Meyer = 8	Figs. 2 and 3, A
		MagMax-mod + Meyer = 10	Figs. 2, A and 3, A
Tuberculosis patients	Urine	CNA + Meyer = 66	Fig. 2
Lung transplant patients	Plasma	CNA + Meyer = 40 [De Vlaminck et al. (12), Burnham et al.(14)]	Fig. 2, B

* Method codes are described in (Table 1).

To calculate the expected MPM, we first evaluated the expected effect of each kit on the host and microbial fragment length profiles by taking the product of the appropriate transfer function and the intrinsic density. The yield was then quantified as the area under the curve of the transformed profile using the bayestestR package (method = “trapezoid”) (16), and the expected MPM was taken as the ratio of microbial to host yield.

### Statistical Analysis and Data Availability

All statistical analyses were performed in R 3.5.0. Boxes in the boxplots indicate the 25th and 75th percentiles, the band in the box represents the median, and whiskers extend to 1.5 x interquartile range (IQR) of the hinge. The sequence data for the kidney transplant recipient cohort was deposited in the NCBI database of Genotypes and Phenotypes (dbGaP, accession number phs001564.v3.p1). The sequence data for the lung transplant cohort was deposited in the NCBI Sequence Read Archive (accession no. PRJNA263522). The sequence data generated in the scope of this study will be deposited in the NCBI Sequence Read Archive.

## Results

### Pre-Analytical Variables Introduce Assay-Specific Biases

Metagenomic cfDNA sequencing assays follow 3 major experimental steps: isolation of the DNA from the biofluid, DNA sequencing library preparation, and DNA sequencing. The efficiency of each of these steps is dependent on the DNA fragment length, and therefore each of these steps introduces fragment length-related biases. The distribution of DNA fragment lengths measured after sequencing is the product of the intrinsic fragment length profile of the original cfDNA sample and the transfer functions of the experimental procedures used, where a transfer function is computed as the yield of the experimental procedure over all fragment lengths. We set out to characterize the transfer functions of 5 different commercial kits for cfDNA isolation, 1 protocol for double-stranded DNA library preparation, and 2 protocols for single-stranded DNA library preparation to better understand the measurement biases they introduce (Table 1).

To create measurement bias models, we mixed isolates of ΦX174 RF I DNA sheared to 5 target lengths to create a DNA sample that exhibited a broad, smooth fragment length profile ranging from 0 to 500 bp (Fig. 1, A). This synthetic sample enabled us to measure the fragment length profile of the sample before and after each experimental procedure via fragment analysis and, from these measurements, the transfer functions of each experimental step (Fig. 1, B).

There were clear differences in the transfer functions of different DNA isolation and library preparation procedures. Three of the DNA isolation kits (CNA, MagMax, and MagMax-mod) exhibited a relatively flat transfer function (10% to 25% yield across all fragment lengths), while the other two kits (Norgen and QiaQuick) displayed a strong bias towards shorter DNA fragments, with yields up to 100% for fragments shorter than 100 bp (Fig. 1, C). The library preparation protocols also displayed distinct transfer functions (Fig. 1, D). The yields of the 2 single-stranded library preparation assays (Meyer and SRSLY) were strongly dependent on fragment length, whereas the yield of the double-stranded protocol (NEB) was relatively insensitive to fragment length. The overall transfer function for each combination of experimental procedures was obtained by taking the product of the individual functions with the transfer function of the sequencing method [obtained from (18)] (Fig. 1, E and F; Supplemental Fig. 1).

### Biases in Pre-Analytical Variables Distort the Measured Fragment Length Distributions

**Fig. 2.**
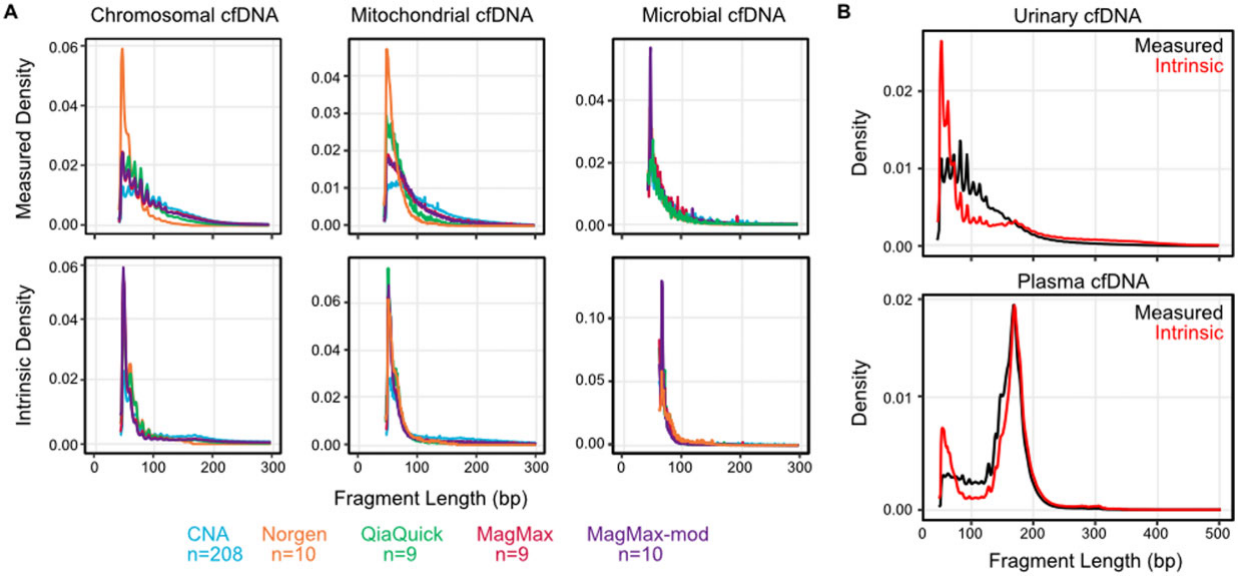
Unskewing fragment length biases introduced by cfDNA sequencing assays reveals the sample-intrinsic fragmentation profiles of cfDNA. (A) The measured fragment length distributions of chromosomal, mitochondrial, and microbial cfDNA (top) converge after deconvolution with the appropriate transfer function, revealing a single underlying distribution (bottom). (B) Measured and sample-intrinsic cfDNA fragmentation profiles for urinary (top) and plasma (bottom) cfDNA.

To assess the length biases introduced in metagenomic cfDNA sequencing by different DNA isolation kits, we measured the fragment length profiles of chromosomal, mitochondrial, and microbial cfDNA in the urine of individuals with urinary tract or tuberculosis infection. We performed paired-end DNA sequencing for a total of 124 urinary cfDNA samples that were prepared using different combinations of cfDNA isolation and library preparation protocols, and analyzed an additional 181 data sets of matched samples that were produced with our reference workflow in the scope of prior studies (1, 11, 12, 14). The measured cfDNA fragment length distributions for chromosomal and mitochondrial cfDNA were dependent on the DNA isolation method employed (Fig. 2, A, top row). The measured fragment length profile of microbial cfDNA was similar for all kits, which we attribute to the highly fragmented nature of microbial cfDNA. This results in a very narrow fragment length distribution that is not markedly distorted by measurement-related length biases.

To obtain the intrinsic fragment length distribution of urinary cfDNA, we corrected the measured distributions with their appropriate transfer function. This resulted in convergence to a single fragment length profile for chromosomal, mitochondrial, and microbial cfDNA (Fig. 2, a, bottom row; Supplemental Tables 1 and 2). Several features of the intrinsic fragment length distributions stand out. First, urinary cfDNA is much shorter than the uncorrected measurements indicate. For example, the combination of MagMax DNA isolation followed by Meyer library preparation and sequencing, gives rise to an estimated 43.4% chromosomal and 34.0% of mitochondrial DNA fragments longer than 100 bp, whereas only 11.7% of the intrinsic chromosomal fragments and 8.5% of the intrinsic mitochondrial fragments are longer than 100 bp. Second, the intrinsic fragment length profile revealed a peak around 167 bp, consistent with the length of DNA in an intact nucleosomal particle, which was not apparent in the uncorrected length profiles (Fig. 2, B, top). We applied the length bias correction model to plasma cfDNA, revealing a dinucleosome peak at 308 bp that was not apparent without bias correction (Fig. 2, B, bottom). We conclude that substantial insight into the biophysical properties of cfDNA in plasma and urine can be obtained by accounting for fragment length biases introduced in cfDNA sequencing protocols.

### Microbial Enrichment is Strongly Correlated with the Isolation Efficiency of Short cfDNA Fragments

**Fig. 3.**
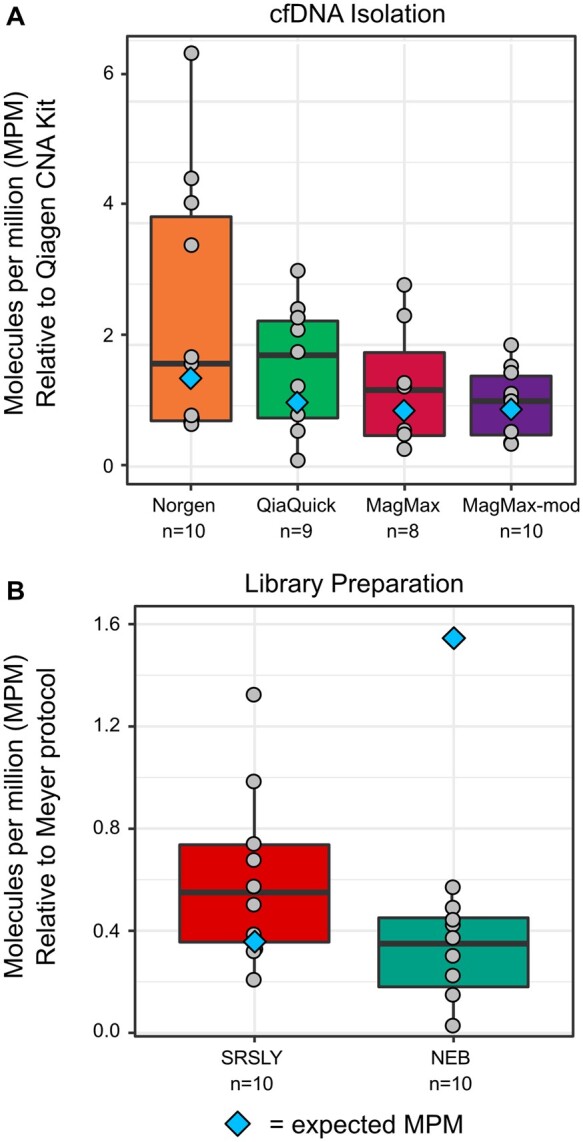
Sensitivity of metagenomic cell-free DNA sequencing is a function of the choice of DNA isolation and library preparation methods. **(**A) Fraction of microbial cfDNA (MPM) for 4 library preparation protocols relative to the CNA kit for cfDNA isolation. (B) Fraction of microbial cfDNA recovered (MPM) for two library preparation protocols relative to the Meyer protocol for library preparation. Blue diamonds indicate the expected MPM based on modeling.

Based on our observation that pre-analytical variables can affect the measured cfDNA fragment length distribution, combined with the fact that different cfDNA classes have distinct fragmentation profiles (8, 14, 30, 31), we hypothesized that the relative yield of microbial cfDNA would depend on the choice of pre-analytical variables. Since we found that microbial cfDNA is more fragmented than host-derived cfDNA, we expected that DNA isolation methods that favor short fragments would be more efficient at recovering microbial cfDNA. To test this idea, we selected samples from 20 patients diagnosed with urinary tract infection by conventional culture (*E.* *coli* = 4, *Pseudomonas aeruginosa* = 2, *Enterococcus faecalis* = 3, *Klebsiella pneumonia* = 2). Two aliquots of each sample were subject to cfDNA isolation using one of the remaining 4 kits followed by library preparation with the Meyer protocol. We used previously established bioinformatics approaches to quantify microbial- and host-specific reads and computed the MPM relative to the CNA kit (11). We found that DNA isolation by the Norgen kit, which favors shorter fragments of DNA, resulted in over 3-fold relative enrichment in MPM (Fig. 3, A), in agreement with expected results computed based on our model, which slightly underestimated the expected MPM. We conclude that the measured transfer functions and bias correction model are predictive of the performance of DNA isolation methods in the recovery of microbial cfDNA.

### Fragment Length Biases Do Not Fully account for the Effects of Library Preparation on Microbial Enrichment

We next experimentally tested the effect of different library preparation procedures on the relative yield of microbial vs host specific cfDNA. We selected samples from 10 patients diagnosed with urinary tract infection by conventional culture (*E.* *coli* = 6, *P.* *aeruginosa* = 1, *K.* *oxytoca* = 1, *E.* *faecalis* = 2). cfDNA was isolated from 2 aliquots per sample using the CNA kit and library preparation was performed using either the NEB or SRSLY kit. We used the experimental transfer functions to unskew the measured fragment length distributions, which did not yield a single, converged fragment length profile (Supplemental Table 3). This observation provided the first indication that fragment length biases alone do not explain the differences in measured fragment length distributions between library preparation protocols.

We calculated the relative number of microbial reads that resulted from the different library preparations and found that the length bias model for library preparation protocols were not predictive (Fig. 3, B): the model predicted that the double-stranded DNA protocol would significantly enrich microbial cfDNA, but in practice both single-stranded library preparation protocols outperformed the double-stranded library preparation. This was the second indication that fragment length biases alone do not explain the differences in measured fragment length distributions between library preparation protocols. We propose that this discrepancy between the length bias model predictions and experimental observations is due to the physical configuration of cfDNA, which is often highly degraded and frequently exists in various states that may contain long, single-stranded DNA overhangs or single-stranded DNA gaps (32), which are difficult to recover via double-stranded DNA ligation. The data therefore suggest that the efficiency of library preparation assays is impacted by both the physical configuration of cfDNA and fragment length biases.

## Discussion

In this study, we show that the measured fragment length distributions of urinary and plasma cfDNA and the recovery of microbial- and host-specific cfDNA are dependent on the choice of pre-analytical variables. Data distortions due to cfDNA isolation can be accounted for by transfer functions which, when applied to measured fragment length distributions, produce a common underlying fragment length distribution. Correction for fragment length biases yields a single distribution that is very short, with a mean fragment length <100 bp for both host- and microbe-specific cfDNA.

The performance and sensitivity of a metagenomic sequencing assay is directly correlated with the microbial enrichment over host reads. The cfDNA isolation transfer functions readily account for these differences, with kits favoring shorter fragments recovering over 3-fold more clinically reported microbial reads per human read. However, the sequencing library preparation protocols do not tell a similar, straightforward story. Fragment length biases only partially account for practical differences in library preparation methods, suggesting that the physical configurations of cfDNA constitute another driving force. Because both single-stranded library preparation protocols outperformed the double-stranded library preparation assay in terms of microbial enrichment, we believe that these differences lie in the sensitivity of each assay to different DNA conformations. The double-stranded library preparation protocol is effective at capturing blunt-ended double-stranded fragments, like the synthetic sample used to characterize the transfer functions, but is insensitive to the gamut of forms that might be found in a cfDNA sample. Single-stranded library preparation methods are more sensitive to the full range of conformations present in cfDNA samples, which may contain nicks, fragments with overhangs, and single-stranded DNA (32). Our study also shows that single-stranded library preparation protocols are more sensitive to highly fragmented and degraded DNA that compose much of the microbial fraction.

Our work underscores the importance of considering multiple biases introduced in the sample preparation workflow to achieve highly sensitive metagenomic cfDNA sequencing assays. It further demonstrates the need for standardization in the liquid biopsy field, particularly in cases where metagenomic cfDNA sequencing is used to guide clinical decisions or where the biophysical properties of cfDNA are used to inform diagnostic technology development. Our findings are relevant for cfDNA applications in prenatal testing and cancer screening, where differences in fragment lengths have been leveraged to improve diagnostic performance (30, 31, 33–38).

## Supplemental Material

Supplemental material is available at *Clinical Chemistry* online.

## Supplementary Material

hvab142_Supplementary_Data

## Employment or Leadership

S.T. Motley, C. Bennett Global Good/Intellectual Ventures Lab; J. Connelly, A. Steadman Global Health Labs

## Consultant or Advisory Role

I. De Vlaminck, Scientific Advisory Board of Karius.

## Stock Ownership

I. De Vlaminck, Karius, Inc.; P. Burnham, Kanvas.

## Honoraria

None declared.

## Expert Testimony

None declared.

## Patents

I. De Vlaminck, inventor of United States Patent No. 10,450,620 and inventor of PCT/US2018/026163; P. Burnham, inventor of PCT/US2018/026163; J.R. Lee, inventor of PCT/US2018/026163 and inventor of US-2020-0048713-A1; D. Dadhania, inventor of PCT/US2018/026163 and patent application W02018187521A2; M. Suthanthiran, inventor of PCT/US2018/026163; A.P. Cheng, inventor of PCT/US2018/026163.

## Other Remuneration

I. De Vlaminck, royalties from Karius, Inc.; J.R. Lee, BioFire Diagnostics, LLC; P. Burnham, travel grant to CVISB workshop.

## Nonstandard Abbreviations

cfDNA: cell-free DNA
MPM: molecules of microbial cfDNA per million reads
