# Supplementary material for: Measurement Biases Distort Cell-Free DNA Fragmentation Profiles and Define the Sensitivity of Metagenomic Cell-Free DNA Sequencing Assays
Source: Clin Chem. 2021 Oct 26;68(1):163–71. doi: 10.1093/clinchem/hvab142 (PMC8718127; doi:10.1093/clinchem/hvab142)
Supplement: hvab142_Supplementary_Data [file hvab142_supplementary_data.pdf]

Supplemental information for:

Measurement biases distort cell-free DNA fragmentation profiles and define the sensitivity of metagenomic cell-free DNA sequencing assays

Authors: Adrienne Chang[1], Omary Mzava[1], Joan S. Lenz[1], Alexandre P. Cheng[1], Philip Burnham[1], S. Timothy Motley[2], Crissa Bennett[2], John T. Connelly[3], Darshana Dadhania[4,5], Manikkam Suthanthiran[4,5], John R. Lee[4,5], Amy Steadman[3], Iwijn De Vlaminck[1*]

Affiliations:

[1]Nancy E. and Peter C. Meinig School of Biomedical Engineering, Cornell University, Ithaca, New York, USA.

[2]Global Good Fund, Intellectual Ventures Lab, Bellevue, WA, USA

[3]Global Health Labs, Bellevue, WA, USA

[4]Division of Nephrology and Hypertension, Department of Medicine, Weill Cornell Medicine, New York, NY, 10065, USA

[5]Department of Transplantation Medicine, New York Presbyterian Hospital–Weill Cornell Medical Center, New York, NY, 10065, USA

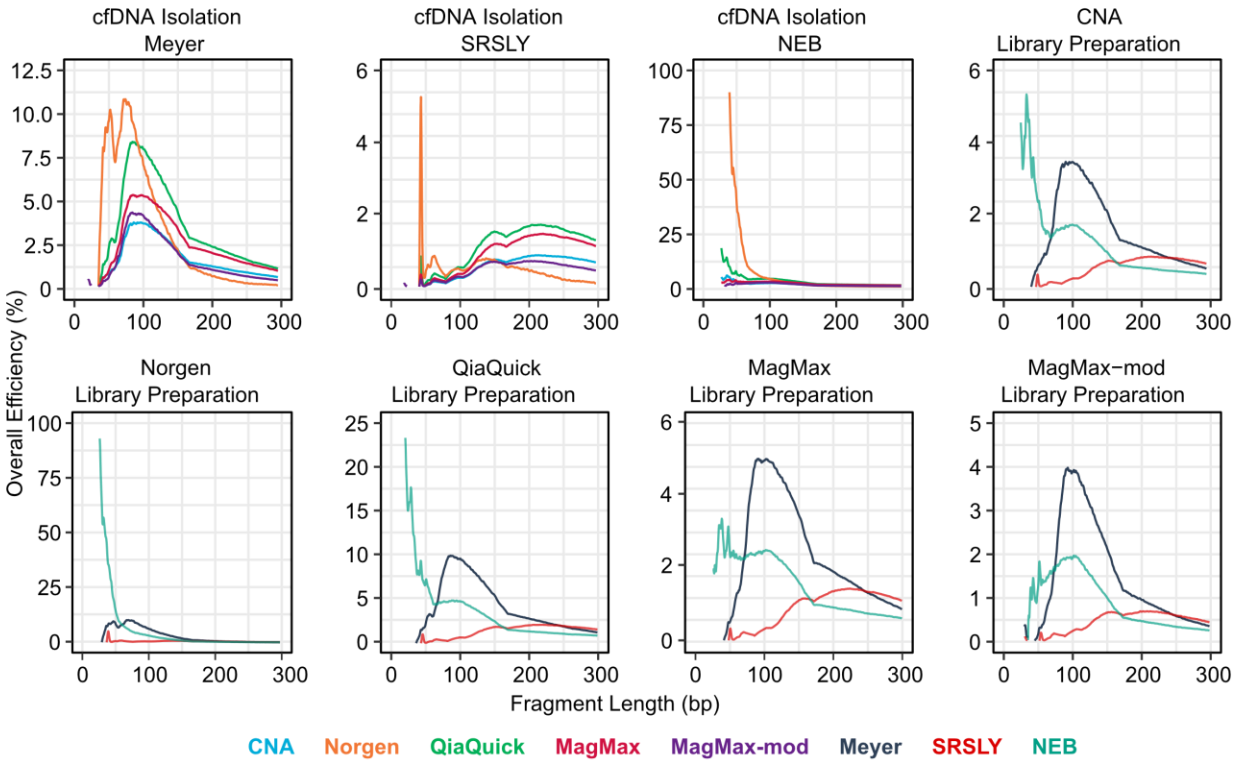

Supplemental Figure 1. Transfer functions for all combinations of cfDNA isolation, library preparation, and sequencing.

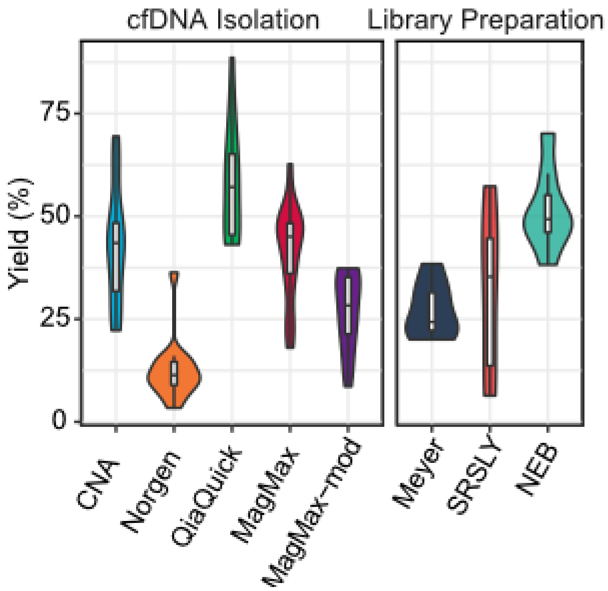

Supplemental Figure 2. Variability in yield for cfDNA isolation and library preparation protocols included in this study (n=15 replicates for each assay).

Supplemental Table 1. Comparison of average fragment lengths measured from different cfDNA isolation kits for chromosomal, mitochondrial, and microbial cfDNA (bp) before and after correction with the transfer function.

		CNA	Norgen	QiaQuick	MagMax	MagMax-mod	Range of Mean Fragment Lengths
**Chromosomal**	**Measured**	101.5	61.5	78.5	90	85.5	61.5 - 101.5
	**Intrinsic**	90.5	61	63.5	63	60	60 - 90.5
**Mitochondrial**	**Measured**	96.5	60.5	68.5	80.5	79.5	60.5 - 96.5
	**Intrinsic**	96.5	60.5	68.5	80.5	79.5	60.5 - 96.5
**Microbial**	**Measured**	99	72.5	91	78	73.5	72.5 - 99
	**Intrinsic**	55.5	59	53	52	50.5	50.5 - 59

Supplemental Table 2. The maximal difference between measures of absolute maximum vertical distance between the cumulative distribution functions of cfDNA isolation kits or library preparation assays.

		**Chromosomal**	**Mitochondrial**	**Microbial**
**cfDNA Isolation**	**Measured**	1.267	1.08	0.256
	**Intrinsic**	0.224	0.200	0.303
	**Fold Change**	5.651	5.387	0.845
**Library Preparation**	**Measured**	0.505	0.302	0.282
	**Intrinsic**	0.419	0.822	0.435
	**Fold Change**	1.205	0.367	0.648

Supplemental Table 3. Comparison of average fragment lengths measured from different library preparation protocols for chromosomal, mitochondrial, and microbial cfDNA (bp) before and after correction with the transfer function.

		Meyer	SRSLY	NEB	Range of Mean Fragment Lengths
**Chromosomal**	**Measured**	101.5	102.5	176	101.5 - 176
	**Intrinsic**	90.5	97.5	214.5	90.5 - 214.5
**Mitochondrial**	**Measured**	96.5	134.5	165.5	96.5 - 165.5
	**Intrinsic**	96.5	134.5	165.5	96.5 - 165.5
**Microbial**	**Measured**	99	123	143	99 - 143
	**Intrinsic**	55.5	57.5	201.5	55.5 - 201.5
